# Direct oral anticoagulants are effective in preventing but not resolving radial artery occlusion: a systematic review and meta-analysis of randomized controlled trials

**DOI:** 10.3389/fcvm.2026.1653388

**Published:** 2026-02-11

**Authors:** Amal A. Alsubaiei, Abdullatif A. Alfehaid, Mooza M. Alzayed, Ali Saad Artam, Osama T. Almutairi, Mohammed S. A. Alajmi, Yaqeen H. Alaraibi, Abdullah A. Alibrahim, Omar F. Alawadhi, Abdullah M. Alharran

**Affiliations:** 1 Kuwait Institute for Medical Specializations, Kuwait City, Kuwait; 2 Royal College of Surgeons in Ireland, Busaiteen, Bahrain; 3 College of Medicine and Medical Sciences, Arabian Gulf University, Manama, Bahrain

**Keywords:** angiography, apixaban, edoxaban, percutaneous coronary intervention (PCI), rivaroxaban, transradial

## Abstract

**Objective:**

This systematic review, including a meta-analysis, aimed to investigate the efficacy and safety of direct oral anticoagulants (DOACs) in preventing and resolving radial artery occlusion (RAO) following transradial artery access.

**Methods:**

We synthesized evidence from randomized controlled trials (RCTs) identified in PubMed, CENTRAL, Scopus, and Web of Science up to May 2025. Using Stata MP v. 17, we employed a fixed-effects model to report dichotomous outcomes, presenting relative risk ratio (RR) with a 95% confidence interval (CI). Trial sequential analysis (TSA) was conducted to investigate the robustness and reliability of the cumulative evidence.

**Results:**

Four RCTs with 970 participants were included. DOACs significantly decreased the incidence of RAO [RR: 0.49, 95% (0.31, 0.79), *p* < 0.001]. However, in terms of RAO resolution, there were no differences between groups [RR: 1.22, 95% CI (0.80, 1.88), *p* = 0.36]. Moreover, there were no differences between groups regarding the incidence of hematoma [RR: 0.56, 95% (0.12, 2.61), *p* = 0.46], minor bleeding [RR: 1.55, 95% (0.72, 3.32), *p* = 0.26], and major bleeding [RR: 2.41, 95% (0.48, 12.25), *p* = 0.36]. There were no incidences of arteriovenous fistula, pseudoaneurysm, or compartment syndrome throughout the trials.

**Conclusion:**

DOACs significantly prevented the incidence of RAO, although TSA indicates that this finding is not yet conclusive. No significant differences were found between groups regarding RAO resolution or the incidence of hematoma, minor bleeding, or major bleeding.

**Systematic Review Registration:**

https://www.crd.york.ac.uk/prospero/, identifier CRD420251063999.

## 1 Introduction

Transradial artery access (TRA) has become the preferred method for most coronary catheterization procedures, exhibiting a reduced incidence of access-related complications compared to the transfemoral approach (1, 2). TRA offers several advantages, including rapid hemostasis, prompt patient mobilization, and a decreased incidence of all-cause mortality, major bleeding, and vascular complications (3). Therefore, the European Society of Cardiology and the American Heart Association recommend TRA as the standard approach, instead of transfemoral access, for all patients undergoing invasive coronary interventions when performed by skilled operators (4, 5). However, in 30%–40% of cases, patients are released without confirmation of radial artery patency. In addition, Doppler examination or plethysmography is used in fewer than 10% of patients, where radial artery patency is assessed (6–8).

Therefore, radial artery occlusion (RAO) has been the most concerning complication following TRA. The rate of RAO in patients who have undergone TRA has varied from less than 1%–33% (1, 9). While often asymptomatic, the potential occurrence of RAO may contraindicate subsequent ipsilateral TRA procedures, thus restricting future radial artery utilization for coronary artery bypass grafting or hemodialysis access (10). The primary pathogenesis of RAO after a TRA procedure is thrombus formation, resulting from the combined effects of endothelial and vascular injury, local hypercoagulability, and decreased blood flow (3, 11, 12). Hence, the significance of sufficient procedural anticoagulation in mitigating early RAO incidence is recognized, yet consensus on the optimal anticoagulation regimen remains lacking (3, 13).

Despite the adoption of best practices such as patent hemostasis, RAO persists in a significant proportion of patients, necessitating the exploration of pharmacological adjuncts (1). Direct oral anticoagulants (DOACs) have emerged as effective and safe oral anticoagulants (OACs) in multiple settings (14–16), and several randomized controlled trials (RCTs) have recently investigated DOACs to prevent and resolve RAO following TRA with promising results (17–20). Therefore, the primary aim of this systematic review, including a meta-analysis, was to evaluate whether the addition of DOACs to standard care is effective and safe for preventing or resolving RAO.

## 2 Methods

### 2.1 Protocol registration

This systematic review was prospectively registered with the International Prospective Register of Systematic Reviews (PROSPERO) (CRD420251063999). The systematic review and meta-analysis were conducted in accordance with the Preferred Reporting Items for Systematic Reviews and Meta-Analyses (PRISMA) guidelines (21) and the Cochrane Handbook for Systematic Reviews of Interventions (22).

### 2.2 Data sources & search strategy

On 17 May 2025, an electronic search was conducted by AbA across the following databases: Web of Science (WOS), PubMed, EMBASE, Scopus, and CENTRAL. The search strategy included the following entry terms: “(anticoagulat* OR “direct oral anticoagulant*” OR DOAC* OR NOAC* OR apixaban OR rivaroxaban OR edoxaban OR dabigatran) AND (“radial artery” OR “radial artery occlusion” OR “radial artery spasm” OR transradial).” Our search strategy employed unrestricted access to most databases; however, Scopus searches were limited to titles and abstracts. Supplementary Table S1 provides a detailed account of the specific search terms and results for each database. A comprehensive review of the reference lists from the included trials was conducted to minimize the risk of excluding relevant studies.

### 2.3 Eligibility criteria

RCTs were included if they met the following PICO criteria: population (P), patients who underwent transradial cardiovascular interventions; intervention (I), DOACs, including rivaroxaban, apixaban, edoxaban, or dabigatran, regardless of the dosing protocol for either prevention of RAO incidence or treatment of established RAO; control (C), no treatment or another anticoagulant; and outcomes (O): primary outcome was the incidence of RAO up to 30 days following procedure and secondary outcomes included established RAO resolution and adverse events (hematoma, minor bleeding, major bleeding, arteriovenous fistula, compartment syndrome, and pseudoaneurysm). Furthermore, our analysis excluded quasi-randomized trials, conference presentations/proceedings, observational studies, *in vitro* research, and review articles.

### 2.4 Study selection

Covidence was used by two independent reviewers (AlA and OA) for thorough screening. After eliminating duplicates, a two-stage evaluation process was employed: initial title and abstract screening, followed by a full-text review of the remaining records. Disagreements were settled through discussion and consensus.

### 2.5 Data extraction

A pilot extraction of relevant publications was conducted to develop an Excel extraction form after obtaining the full texts of eligible publications. The form was structured into three sections: (1) summary characteristics of the included trials (study ID, country, study design, sample size, treatment protocols, main inclusion criteria, and follow-up duration); (2) baseline characteristics of the included participants (age, gender, and comorbidities); and (3) outcomes [the incidence of RAO up to 30 days following the procedure, established RAO resolution, and the adverse events (hematoma, minor bleeding, major bleeding, arteriovenous fistula, compartment syndrome, and pseudoaneurysm)]. Two reviewers independently extracted the data (AlA and OA), resolving disagreements through discussion with a senior author. Dichotomous data were documented as event rates, whereas continuous data were summarized using means and standard deviations.

### 2.6 Risk of bias and certainty of evidence

The revised Cochrane Collaboration tool for RCTs (ROB 2) was employed to evaluate the risk of bias in the included studies (23). Two reviewers (M.S.A and Y.H.A.) independently assessed each study, examining domains such as selection bias, performance bias, reporting bias, attrition bias, and other potential sources of bias. Disagreements were resolved through a consensus-building process. In addition, the certainty of the evidence was evaluated using the Grading of Recommendations Assessment, Development, and Evaluation (GRADE) framework (24, 25). This assessment considered factors such as inconsistency, imprecision, indirectness, publication bias, and risk of bias. Each factor was individually analyzed, with decisions clearly justified and documented. Any discrepancies in the evaluation were addressed through discussion to reach a mutually agreed-upon resolution.

### 2.7 Statistical analysis

Data analysis was conducted using Stata MP, version 17 (StataCorp). Dichotomous outcomes were analyzed using the risk ratio (RR), with 95% confidence intervals (CIs). The fixed-effect model was the primary approach; however, a random-effects model was adopted in the presence of significant heterogeneity. Heterogeneity among the included studies was assessed using the chi-squared test and the *I*-squared statistic (*I*^2^), with a *p*-value of <0.1 for the chi-square test and an *I*^2^ value of 50% or higher indicating significant heterogeneity. To account for the observed clinical heterogeneity and investigate the stability of the pooled results, we conducted leave-one-out sensitivity analysis. Publication bias was not assessed, as all evaluated outcomes included fewer than 10 randomized controlled trials (RCTs) (26). Finally, trial sequential analysis (TSA) was conducted to assess the robustness and conclusiveness of the meta-analytic results. TSA considered the size of the information and the cumulative *z*-curve to determine the sufficiency and robustness of the available evidence. Boundary controls were established to manage the risks associated with Type I and Type II errors. TSA was conducted using the trial sequential analysis software (27).

## 3 Results

### 3.1 Search results and study selection

**Figure 1:**
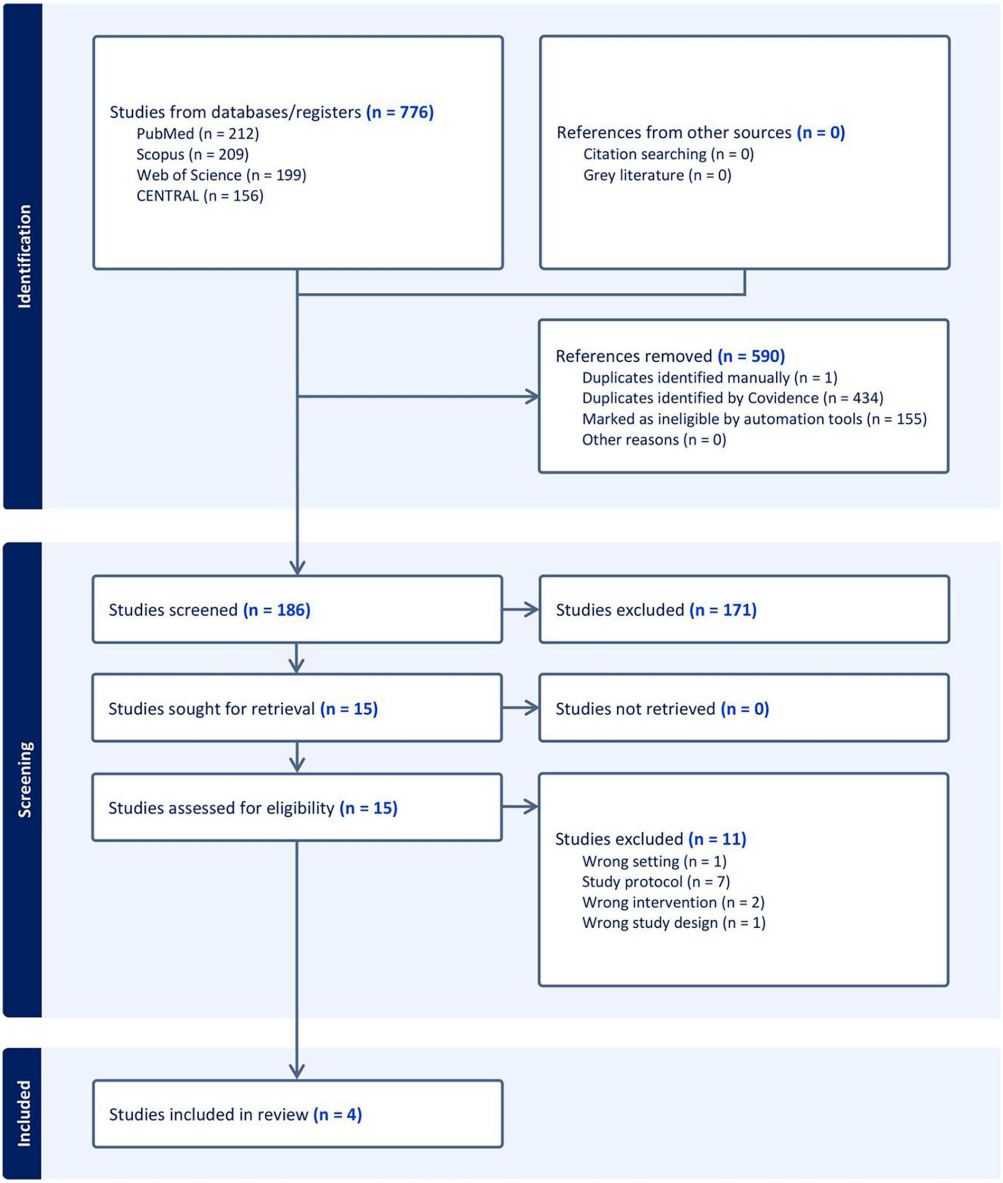
PRISMA flow chart of the screening process.

Following a literature search, 776 studies were identified. Covidence automatically removed 590 irrelevant records, leaving 186 records to be screened. Following title and abstract screening, 171 studies were excluded for failing to meet the inclusion criteria, leaving 15 full-text articles for further assessment. Of these, 11 studies were excluded (Supplementary Table S2), leaving four RCTs (17–20) to be included in qualitative and quantitative analyses (Figure 1).

### 3.2 Characteristics of included studies

Four trials with a total of 970 participants were included (17–20). Two trials investigated DOACs for RAO prophylaxis (17, 19) and another two used DOACs to treat established RAO (18, 20). Three trials used rivaroxaban (17–19), and one used apixaban (20). No eligible RCTs evaluating dabigatran or edoxaban were identified during the search process. The control group consisted of watchful waiting with no treatment in three trials (17–19), and one trial used enoxaparin as a control (20). Further details on the treatment protocols, study design, and patient information are provided in Tables 1, 2.

**Table 1:** Summary characteristics of the included RCTs.

Study ID	Study design	Country	Total participants	DOACs		Control	Treatment/prophylaxis	RAO diagnostic method	Timing of RAO assessment	Definition of RAO	Primary outcome	Follow-up duration
				Drug	Dosing protocol							
Amirpour et al. (20)	RCT	Iran	30	Apixaban	2.5 mg/twice daily	No treatment	Treatment	Doppler ultrasonography	Baseline: symptomatic onset postprocedure; Follow-up: 30 days postprocedure	Absence of forward blood flow detected in triphasic waves	Radial artery recanalization	30 days
Hammami et al. (RIVARAD) (17)	RCT	Tunisia	521	Rivaroxaban	10 mg for 7 days after the procedure	No treatment	Prevention	Doppler ultrasonography	Follow-up: 30 days postprocedure	No antegrade flow signal	Rate of radial artery occlusion at 30 days	30 days
Liang et al. (RESTORE) (19)	RCT	China	382	Rivaroxaban	10 mg for 7 days after the procedure	Placebo	Prevention	Doppler ultrasonography	Screening: 24 h postprocedure; Follow-up: 30 days postprocedure	No antegrade flow signal	Rate of radial artery occlusion at 24 h	30 days
Maadani et al. (18)	RCT	Iran	37	Rivaroxaban	15 mg twice daily for 21 days, followed by 20 mg daily for 7 days	Enoxaparin (1 mg/kg twice daily) for 28 days	Treatment	Doppler ultrasonography	Screening: Within 24 h postprocedure; Follow-up: 28 days postrandomization	Complete absence of blood flow (total thrombotic occlusion)	Radial artery recanalization	28 days

RAO, radial artery occlusion; RCT, randomized controlled trial; DOACs, direct oral anticoagulants.

**Table 2:** Baseline characteristics of the participants.

Study ID	Number of patients		Age (years), mean (SD)		Gender (male), *N* (%)		Comorbidities							
	DOACs	Control	DOACs	Control	DOACs	Control	HTN		DM		Smoking		Stoke	
							DOACs	Control	DOACs	Control	DOACs	Control	DOACs	Control
Amirpour et al. (20)	15	15	59.14 (12.85)	59.73 (11.43)	12 (80)	6 (40)	8 (53.3)	4 (26.7)	6 (40)	4 (26.7)	6 (40)	1 (6.7)	NA	NA
Hammami et al. (RIVARAD) (17)	259	262	59.41 ± 10	60.1 ± 9	185 (71.4)	167 (63.7)	137 (52.9)	147 (56.1)	114 (44)	137 (52.3)	135 (52.1)	115 (43.9)	NA	NA
Liang et al. (RESTORE) (19)	191	191	64.3 ± 10.1	64.0 ± 10.0	117 (61.3)	126 (66.0)	131 (68.6)	126 (66.0)	60 (31.4)	58 (30.4)	65 (34.0)	69 (36.1)	8 (4.2)	7 (3.7)
Maadani et al. (18)	20	17	54.5 ± 15.2	56.0 ± 8.5	3 (15)	8 (47.1)	11 (55.0)	11 (64.7)	6 (30.0)	5 (29.4)	NA	NA	NA	NA

DOACs, direct oral anticoagulants; SD, standard deviation; *N*, number of patients; HTN, hypertension; DM, diabetes mellitus.

### 3.3 Risk of bias and certainty of evidence

**Figure 2:**
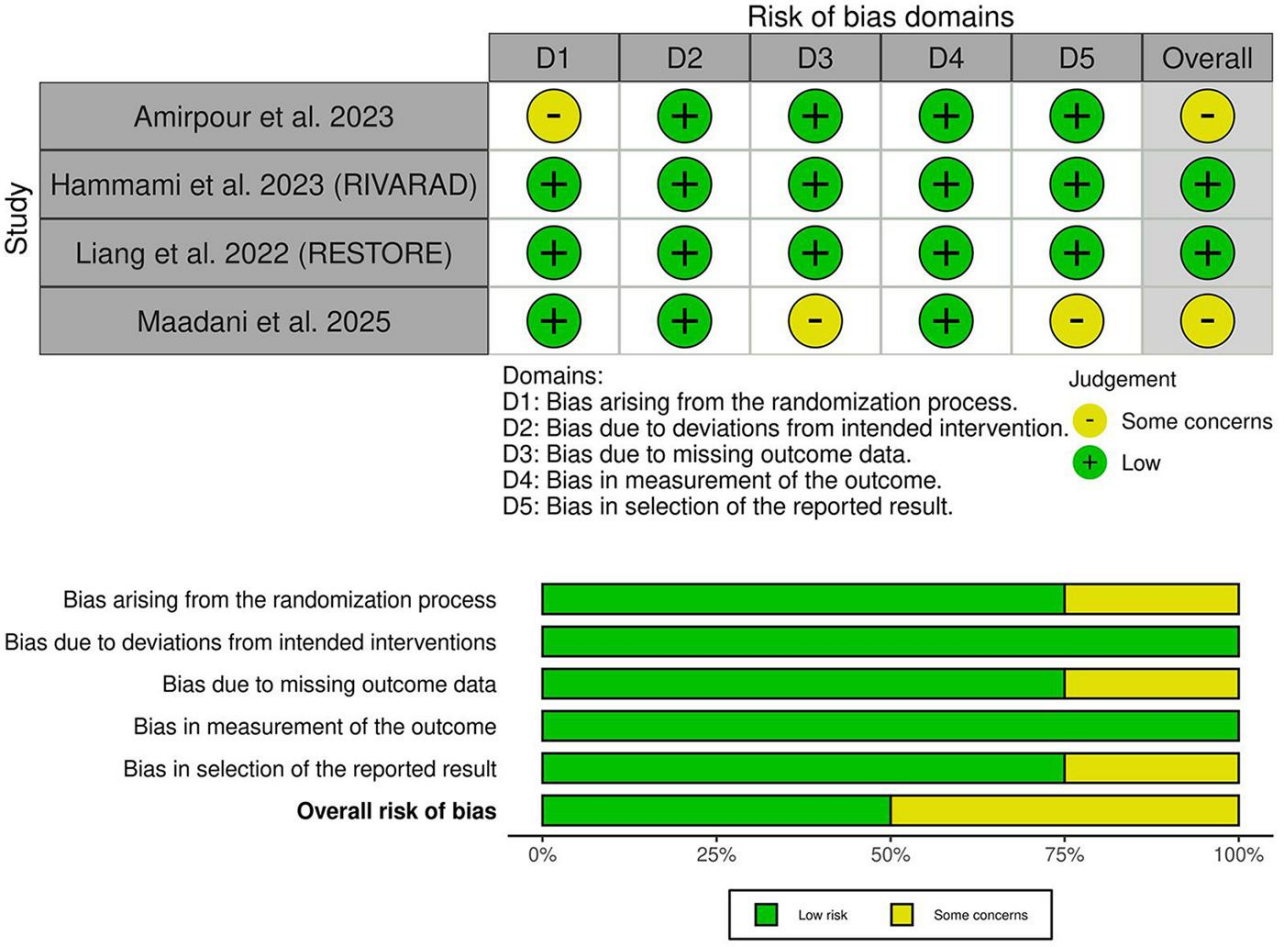
Quality assessment of risk of bias in the included trials. The upper panel presents a schematic representation of risks (low = green, some concerns = yellow, high = red) for specific types of biases in the studies reviewed. The lower panel presents risks (low = green, some concerns = yellow, high = red) for the subtypes of biases of the combination of studies included in this review.

Two trials demonstrated an overall low risk of bias (17, 19), while two trials showed some concerns of bias (18, 20) (Figure 2). Amirpour et al. showed some concerns regarding selection bias due to a lack of information about the randomization process (20). In contrast, Maadani et al. raised concerns about attrition bias due to the 15% drop in patients in the control group without clear justification (18). The GRADE certainty of evidence is summarized in Table 3.

**Table 3:** GRADE evidence profile.

Certainty assessment							Summary of findings				
Participants (studies) follow-up	Risk of bias	Inconsistency	Indirectness	Imprecision	Publication bias	Overall certainty of evidence	Study event rates (%)		Relative effect (95% CI)	Anticipated absolute effects	
							With (Control)	With (DOACs)		Risk with (Control)	Risk difference with (DOACs)
Radial artery occlusion											
835 (2 RCTs)	Not serious	Not serious	Not serious	Very serious	None	⊕⊕◯◯ Low^a^	52/419 (12.4%)	24/416 (5.8%)	**RR 0.49** (0.31 to 0.79)	52/419 (12.4%)	63 fewer per 1,000 (from 86 fewer to 26 fewer)
Radial artery occlusion resolution											
100 (3 RCTs)	Not serious	Not serious	Not serious	Extremely serious^a^	None	⊕◯◯◯ Very low^a^	24/52 (46.2%)	31/48 (64.6%)	**RR 1.22** (0.80 to 1.88)	24/52 (46.2%)	102 more per 1,000 (from 92 fewer to 406 more)
Hematoma											
835 (2 RCTs)	Not serious	Not serious	Not serious	Extremely serious^a^	None	⊕◯◯◯ Very low^a^	4/419 (1.0%)	2/416 (0.5%)	**RR 0.56** (0.12 to 2.61)	4/419 (1.0%)	4 fewer per 1,000 (from 8 fewer to 15 more)
Minor bleeding											
940 (3 RCTs)	Not serious	Not serious	Not serious	Very serious^a^	None	⊕⊕◯◯ Low^a^	10/470 (2.1%)	16/470 (3.4%)	**RR 1.55** (0.72 to 3.32)	10/470 (2.1%)	12 more per 1,000 (from 6 fewer to 49 more)
Major bleeding											
940 (3 RCTs)	Not serious	Not serious	Not serious	Extremely serious^a^	None	⊕◯◯◯ Very low^a^	1/470 (0.2%)	4/470 (0.9%)	**RR 2.41** (0.48 to 12.25)	1/470 (0.2%)	3 more per 1,000 (from 1 fewer to 24 more)

CI, confidence interval; RR, risk ratio; explanations.

Bold values indicate statistically significant results.

a A wide confidence interval with a low number of events.

### 3.4 Primary outcome: radial artery occlusion incidence

**Figure 3:**
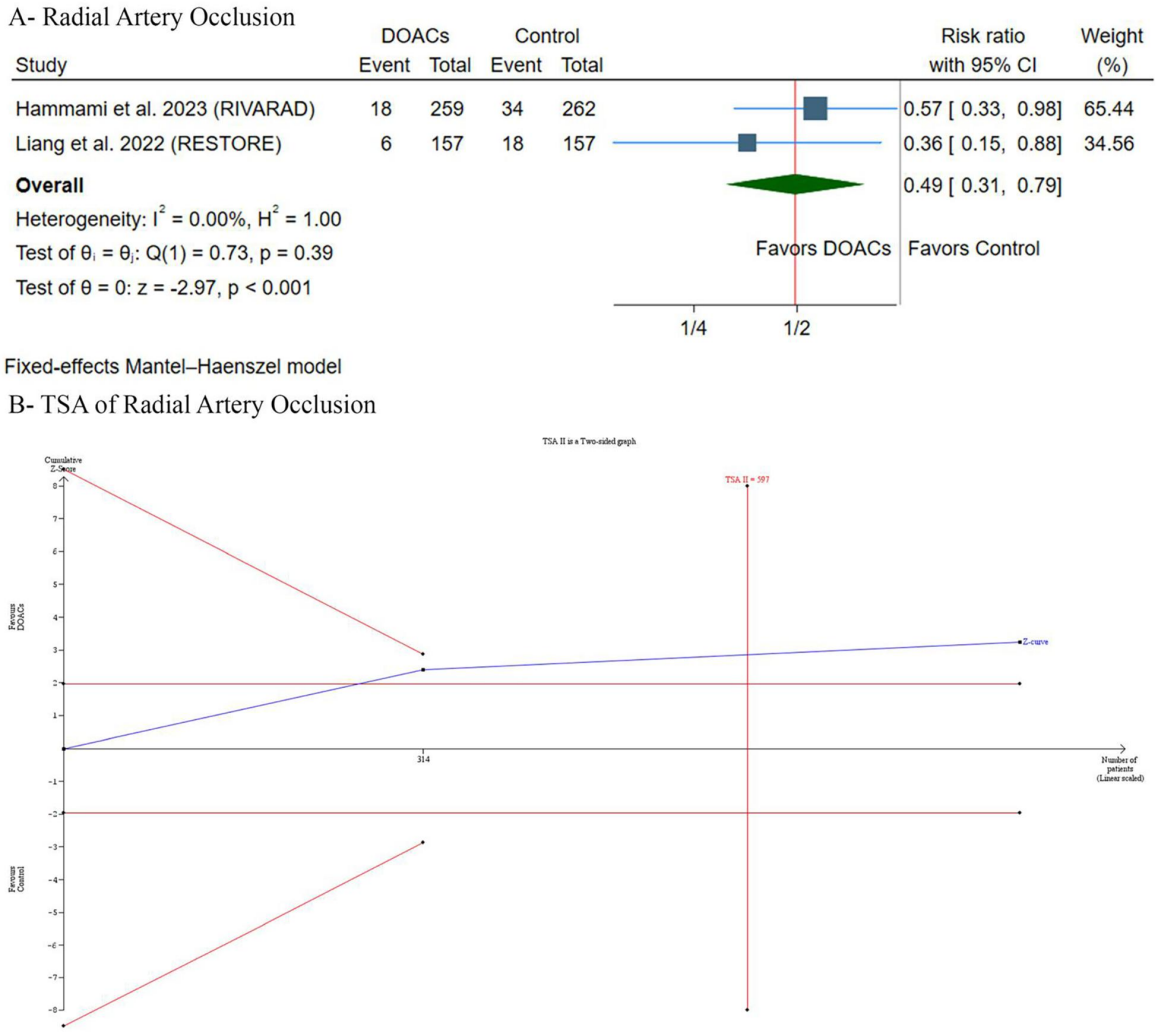
**(A)** Forest plot of radial artery occlusion incidence comparing. DOACs versus control. **(B)** Trial sequential analysis of radial artery occlusion incidence. RR, risk ratio; CI, confidence interval.

DOACs significantly decreased the incidence of RAO [RR: 0.49, 95% (0.31, 0.79), *p* < 0.001] (Figure 3A). Pooled studies were homogeneous (*p* = 0.39, *I*^2^ = 0%). TSA suggested a trend favoring DOACs, as the *Z*-curve crossed the conventional statistical significance boundary. However, it did not cross the more conservative TSA-adjusted boundary, and the required information size of participants had not yet been reached (Figure 3B). Therefore, while current evidence suggests a benefit with DOACs, the findings are not yet conclusive according to the TSA, and further research may be needed to strengthen the findings and mitigate the risk of random errors in this cumulative analysis.

### 3.5 Secondary outcomes

#### 3.5.1 RAO resolution

**Figure 4:**
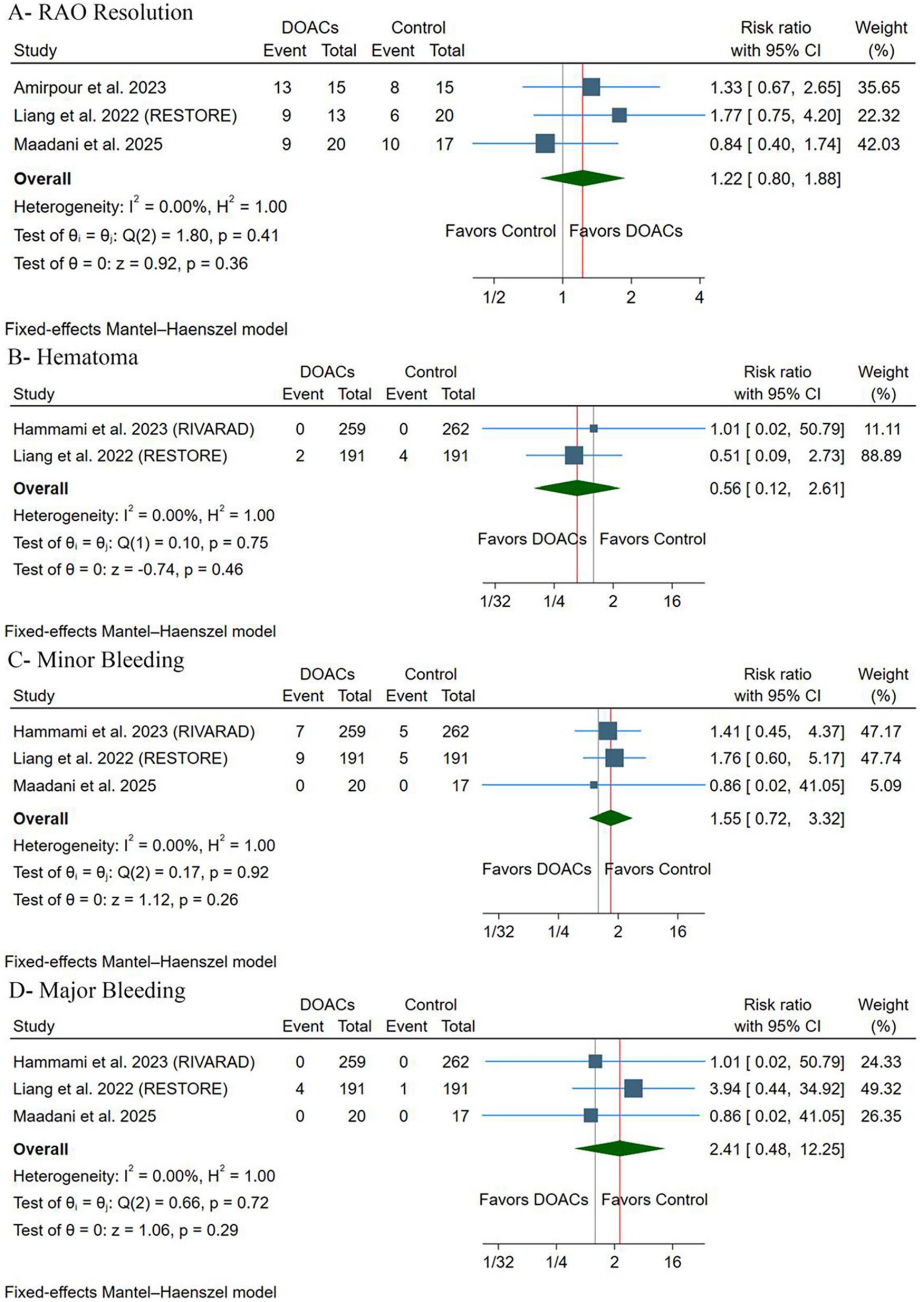
**(A)** Radial artery occlusion resolution. **(B)** Hematoma. **(C)** Minor bleeding. **(D)** Major bleeding. RR, risk ratio; CI, confidence interval.

There were no differences observed between groups [RR: 1.22, 95% (0.80, 1.88), *p* = 0.36] (Figure 4A). Pooled studies were homogeneous (*p* = 0.41, *I*^2^ = 0%). Leave-one-out sensitivity analysis revealed that the results were stable, with no significant statistical difference observed across exclusion scenarios (Supplementary Figure S1).

#### 3.5.2 Adverse events

There were no differences between groups regarding the incidence of hematoma [RR: 0.56, 95% (0.12, 2.61), *p* = 0.46] (Figure 4B), minor bleeding [RR: 1.55, 95% (0.72, 3.32), *p* = 0.26] (Figure 4C), or major bleeding [RR: 2.41, 95% (0.48, 12.25), *p* = 0.36] (Figure 4D). Pooled studies were homogeneous in terms of hematoma (*p* = 0.75, *I*^2^ = 0%), minor bleeding (*p* = 0.92, *I*^2^ = 0%), and major bleeding (*p* = 0.72, *I*^2^ = 0%). There were no cases of arteriovenous fistula, pseudoaneurysm, or compartment syndrome throughout the included trials. Leave-one-out sensitivity analysis revealed that the results were stable, with no significant statistical difference observed in all exclusion scenarios for minor bleeding (Supplementary Figure S2) and major bleeding (Supplementary Figure S3).

## 4 Discussion

After synthesizing evidence from four RCTs involving 970 patients, DOACs were effective in preventing the incidence of RAO after TRA procedures but not in resolving the already established RAO. In addition, DOACs were well tolerated, with no difference compared to the control groups regarding the incidence of hematoma and bleeding, and no reported cases of arteriovenous fistula, pseudoaneurysm, or compartment syndrome across the included trials.

A significant risk factor for RAO is thrombus formation after endothelial injury with reduced blood flow after sheath and catheter insertion (12). Repeated radial artery cannulation may lead to intimal hyperplasia and increased intima-media thickness, thereby contributing to adverse arterial wall remodeling and an increased risk of RAO (28). Stagnant blood during hemostasis can lead to thrombus formation, potentially causing vessel occlusion (1). Reducing compression time and sheath size minimizes the risk of RAO by lessening endothelial injury (29). Maintaining the radial artery's long-term function after transradial artery catheterization is crucial for optimal patient outcomes. Therefore, it is crucial to urgently investigate effective preventive and abortive measures of RAO.

The contradictory effect of DOACs—preventing but not resolving RAO—is worth further investigation. RAO can result from endothelial damage and reduced blood flow caused by sheath and catheter insertion, as well as procedural manipulations, which activate the coagulation cascade, leading to the formation of thrombi and causing radial artery thrombosis (30). Artery compression by local edema or hematoma is another contributing factor (31). Early administration of DOACs may prevent this cascade of events, explaining their preventative efficacy. However, DOACs may need a more prolonged period to resolve an established RAO. Liang et al. reported that the 24 h RAO rate was similar in the rivaroxaban and control groups, indicating that a single 10 mg postprocedural dose of rivaroxaban may be insufficient to prevent early RAO (19). This might be because the RAO occurred before the rivaroxaban took effect, since it was given after the bleeding had stopped. Therefore, the thrombus persisted due to the insufficient duration of rivaroxaban's effect, resulting in a lower radial artery recanalization rate (19).

Moreover, baseline differences in RAO risk factors may have contributed to this effect. Modifiable patient risk factors include diabetes mellitus, hyperlipidemia, peripheral artery disease, multivessel cardiovascular disease, and kidney diseases (3). Unmodifiable patient risk factors include ethnicity (South Asian descent), female gender, low body mass index, older age, reduced radial artery diameter, and prior catheterizations at the same arterial access site (3). In addition, modifiable procedural factors include insufficient anticoagulation, extended procedure times, multiple catheter use, radial artery spasm, repeated arterial punctures, and prolonged or occlusive postoperative hemostasis (3). Larger sheaths and mismatches between sheath and artery size (indicated by a high sheath-to-artery ratio) can damage the vessel wall, increasing the risk of RAO (10, 29).

Hammami et al. found that smoking, the female sex, and a history of TRA punctures predicted the occurrence of RAO (17). Among smoking women in their cohort with a history of TRA puncture, RAO occurred in 50%. Contributing factors may include the smaller radial artery diameter in women compared to men, the known prothrombotic effects of tobacco use, and the possibility of repeated trauma to the artery. However, their multivariate logistic regression analysis showed that a week of postoperative rivaroxaban (10 mg daily) and the heparin given during the procedure independently predicted a lower incidence of 1-month RAO, with no adverse bleeding observed (17). On the other hand, none of the factors considered—age, sex, BMI, smoking, diabetes, hypertension, statin use, antiplatelet regimen (single or dual), intervention duration, and radial artery diameter—were associated with RAO resolution as reported by Amirpour et al. (20). Nevertheless, most research to date has focused on factors associated with the incidence of RAO, rather than its resolution.

Our pooled analysis has the following limitations: First, we included a limited number of RCTs with a small number of patients, given the context of the overall number of patients undergoing TRA; hence, our results require further confirmation. Second, the geographical representation of the included sample is limited to Asia, further limiting the generalizability of our findings. Third, due to limited data, we could not conduct a meta-regression analysis to investigate the potential effect of different confounders on our results. Fourth, GRADE assessment and TSA indicated that current evidence remains imprecise, warranting careful interpretation of our findings.

In terms of clinical implications, our results suggest that a short-term prophylactic course of DOACs could be a valuable adjunctive strategy in routine practice. Both the RIVARAD and RESTORE trials utilized a regimen of Rivaroxaban 10 mg daily for 7 days following the procedure, demonstrating a significant reduction in 30-day RAO without an increase in major bleeding events (17, 19). This strategy could be especially beneficial for patients at high risk, who are likely to experience occlusion, despite following best practices. For example, Hammami et al. identified female sex, smoking, and previous transradial puncture as independent predictors of RAO (17). Therefore, in cases with difficult patent hemostasis, or for high-risk groups such as those with small radial arteries or smokers, clinicians may consider a short period of low-dose DOACs following discharge to decrease the risk of vessel loss.

Further large-scale RCTs are warranted to investigate the following: First, the optimal DOAC regimen remains unclear. Additional research is necessary to determine the safety and efficacy of a brief, high-dose DOAC regimen. The comparative safety and efficacy of DOACs versus other anticoagulants, such as low-molecular-weight heparin, remain unclear. Second, future trials should investigate the efficacy of DOACs in conjunction with other preventive measures, such as nitroglycerin administration, maintaining a sheath-to-artery ratio of less than one, and prophylactic ipsilateral compression of the ulnar artery (3). Third, the factors that may confound the use of DOACs in RAO resolution remain unclear, warranting further investigation.

## 5 Conclusion

This meta-analysis suggests that DOACs are a promising adjunctive strategy for reducing the incidence of RAO. However, generalization to all radial procedures is premature, as these findings, based on GRADE assessment and TSA, require further confirmation. Moreover, due to the limited number of RCTs, the evidence remains uncertain, making it difficult to draw a definitive conclusion regarding the efficacy of DOACs in resolving established RAO.

## Data Availability

The original contributions presented in the study are included in the article/Supplementary Material; further inquiries can be directed to the corresponding author.
