# Supplementary material for: Direct oral anticoagulants are effective in preventing but not resolving radial artery occlusion: a systematic review and meta-analysis of randomized controlled trials
Source: Front Cardiovasc Med. 2026 Feb 11;13:1653388. doi: 10.3389/fcvm.2026.1653388 (PMC12932602; doi:10.3389/fcvm.2026.1653388)
Supplement: Supplementary file 1 [file Datasheet1.docx]

**Supplementary Materials:**

**Contents:**

**Tables.**Table S1: Search strategy.
Table S2: Excluded records during full-text screening.

**Figures.**Figure S1: Leave-one-out sensitivity analysis of radial artery occlusion resolution.
Figure S2: Leave-one-out sensitivity analysis of minor bleeding.
Figure S3**:** Leave-one-out sensitivity analysis of major bleeding.

Table S1: Search Strategy.

| Database | Search Terms | Search Field | Search Results |
| --- | --- | --- | --- |
| PubMed | (anticoagulat* OR “direct oral anticoagulant*” OR DOAC* OR NOAC* OR Apixaban OR Rivaroxaban OR edoxaban OR dabigatran) AND (“radial artery” OR “radial artery occlusion” OR “radial artery spasm” OR transradial) | All Fields | 212 |
| Cochrane | (anticoagulat* OR “direct oral anticoagulant*” OR DOAC* OR NOAC* OR Apixaban OR Rivaroxaban OR edoxaban OR dabigatran) AND (“radial artery” OR “radial artery occlusion” OR “radial artery spasm” OR transradial) | All Text | 78 |
| WOS | (anticoagulat* OR “direct oral anticoagulant*” OR DOAC* OR NOAC* OR Apixaban OR Rivaroxaban OR edoxaban OR dabigatran) AND (“radial artery” OR “radial artery occlusion” OR “radial artery spasm” OR transradial) | All Fields | 199 |
| SCOPUS | TITLE-ABS ( ( anticoagulat* OR "direct oral anticoagulant*" OR doac* OR noac* OR apixaban OR rivaroxaban OR edoxaban OR dabigatran ) AND ( "radial artery" OR "radial artery occlusion" OR "radial artery spasm" OR transradial ) ) | Title, Abstract | 209 |

Table S2: Excluded records during full-text screening.

| Title | DOI | Study ID | Exclusion reason |
| --- | --- | --- | --- |
| Low-molecular-weight heparin in radial artery occlusion treatment: The LOW-RAO randomized study | 10.2217/fca-2021-0067 | Didagelos 2022 | Wrong intervention |
| Distal access and procedural anticoagulation to prevent radial artery occlusion after coronary angiography â€“ the randomised RAPID trial | 10.4244/EIJ-D-24-00846 | Stiermaier 2025 | Wrong intervention |
| Rationale and Design of the Rivaroxaban Post-Transradial Access for the Prevention of Radial Artery Occlusion Trial (CAPITAL-RAPTOR) | 10.1136/bmjopen-2022-070720 | DiSanto 2023 | Study protocol |
| Safety of radial coronary angiography with uninterrupted direct-acting oral anticoagulant treatment | 10.5543/tkda.2018.82830 | Ã–z 2019 | Wrong study design |
| Efficacy analysis of Short-Term Postoperative Use of Rivaroxaban to Treat Radial Artery Occlusion After Transradial Coronary Procedure | ChiCTR2500097763 2025 | | Study protocol |
| Effect of Rivaroxaban in Radial Artery Occlusion Treatment After Cardiac Catheterization | NCT06812455 2025 | | Study protocol |
| Rivaroxaban Post-Transradial Access for the Prevention of Radial Artery Occlusion (CAPITAL-RAPTOR) | NCT05399277 2022 | | Study protocol |
| RIVARAD study | PACTR202110633346282 2021 | | Study protocol |
| Strategies to Maintain Radial Artery Patency Following Diagnostic Coronary Angiography - Subgroup of Patients on Oral Anticoagulants | NCT04362020 2020 | | Study protocol |
| Effect of rivaroxaban in radial artery thrombosis | IRCT20200111046084N1 2020 | | Study protocol |
| Anticoagulation profile of high-dose vs. standard-dose enoxaparin for percutaneous coronary intervention | Liu 2020 | | Wrong setting |


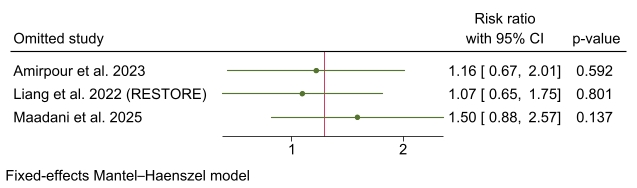


Figure S1: Leave-one-out sensitivity analysis of radial artery occlusion resolution.


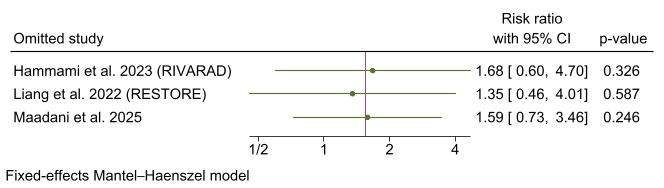


Figure S2: Leave-one-out sensitivity analysis of minor bleeding.


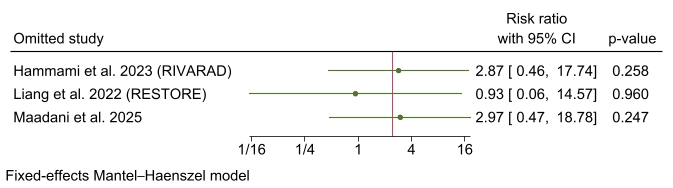


Figure S3: Leave-one-out sensitivity analysis of major bleeding.
